# Five-Year Antimicrobial Resistance Patterns of Urinary *Escherichia coli* at an Australian Tertiary Hospital: Time Series Analyses of Prevalence Data

**DOI:** 10.1371/journal.pone.0164306

**Published:** 2016-10-06

**Authors:** Oyebola Fasugba, Brett G. Mitchell, George Mnatzaganian, Anindita Das, Peter Collignon, Anne Gardner

**Affiliations:** 1 Faculty of Health Sciences, Australian Catholic University, Australian Capital Territory, Australia; 2 Faculty of Arts, Nursing and Theology, Avondale College of Higher Education, Wahroonga, New South Wales, Australia; 3 College of Science, Health and Engineering, La Trobe Rural Health School, La Trobe University, Victoria, Australia; 4 Australian Capital Territory Pathology, Canberra Hospital and Health Services, Garran, Australian Capital Territory, Australia; 5 Medical School, Australian National University, Acton, Australian Capital Territory, Australia; Ross University School of Veterinary Medicine, SAINT KITTS AND NEVIS

## Abstract

This study describes the antimicrobial resistance temporal trends and seasonal variation of *Escherichia coli* (*E*. *coli)* urinary tract infections (UTIs) over five years, from 2009 to 2013, and compares prevalence of resistance in hospital- and community-acquired *E*. *coli* UTI. A cross sectional study of *E*. *coli* UTIs from patients attending a tertiary referral hospital in Canberra, Australia was undertaken. Time series analysis was performed to illustrate resistance trends. Only the first positive *E*. *coli* UTI per patient per year was included in the analysis. A total of 15,022 positive cultures from 8724 patients were identified. Results are based on 5333 first *E*. *coli* UTIs, from 4732 patients, of which 84.2% were community-acquired. Five-year hospital and community resistance rates were highest for ampicillin (41.9%) and trimethoprim (20.7%). Resistance was lowest for meropenem (0.0%), nitrofurantoin (2.7%), piperacillin-tazobactam (2.9%) and ciprofloxacin (6.5%). Resistance to amoxycillin-clavulanate, cefazolin, gentamicin and piperacillin-tazobactam were significantly higher in hospital- compared to community-acquired UTIs (9.3% versus 6.2%; 15.4% versus 9.7%; 5.2% versus 3.7% and 5.2% versus 2.5%, respectively). Trend analysis showed significant increases in resistance over five years for amoxycillin-clavulanate, trimethoprim, ciprofloxacin, nitrofurantoin, trimethoprim-sulphamethoxazole, cefazolin, ceftriaxone and gentamicin (P<0.05, for all) with seasonal pattern observed for trimethoprim resistance (augmented Dickey-Fuller statistic = 4.136; P = 0.006). An association between ciprofloxacin resistance, cefazolin resistance and ceftriaxone resistance with older age was noted. Given the relatively high resistance rates for ampicillin and trimethoprim, these antimicrobials should be reconsidered for empirical treatment of UTIs in this patient population. Our findings have important implications for UTI treatment based on setting of acquisition.

## Introduction

Urinary tract infections (UTIs) are predominantly bacterial infections affecting people both in the community and in hospitals [1]. Over 80% are caused by *Escherichia coli (E*. *coli)*, a Gram negative bacillus [2]. Data from the combined National Ambulatory Health Care Surveys in the United States (US) for 2009–2010 showed that UTIs accounted for approximately 9.8 million visits to ambulatory care settings such as primary care, outpatient and emergency departments [3]. Visits due to UTI were estimated to be 0.8% of all ambulatory care visits [3]. In Australia, national data on UTI are unavailable but recent estimates from 82 hospitals and 17 aged care facilities reported a point prevalence of 1.4% and 1.5% respectively for healthcare associated UTIs [4].

While UTIs are a major infection burden globally, the growing problem of antimicrobial resistance (AMR) can result in treatment failures and increased cost of healthcare [5]. There is evidence to show that the AMR pattern of urinary *E*. *coli* is increasing [6]. In Switzerland, an analysis of urinary *E*. *coli* specimens obtained from a university hospital from 1997 to 2007 found an increasing trend in resistance to trimethoprim/sulfamethoxazole, ciprofloxacin and amoxycillin/clavulanic acid (from 17.4% to 21.3%, 1.8% to 15.9%, and 9.5% to 14.5%, respectively) [6]. The Australian Group on Antimicrobial Resistance (AGAR) which undertakes AMR prevalence surveys within Australia also noted a gradual rise in overall percentage of *E*. *coli* strains resistant to beta-lactam antibiotics and ciprofloxacin [7]. From 2009 to 2011, resistance of hospital-onset *E*. *coli* isolates to ampicillin and ciprofloxacin increased from 48% to 51% and 8% to 11% respectively [7]. Furthermore, the resistance rates of urinary *E*. *coli* to various antimicrobials show large inter-country variability [8]. Only a few studies have shown that *E*. *coli* resistance rates differ for hospital-acquired and community-acquired UTIs [9–11]. Measuring and comparing the levels of AMR in both hospital- and community-acquired UTIs is essential because although effects of AMR are mainly felt in healthcare facilities, the greatest use of antimicrobials occurs in the community [12]. Comparing resistance rates in hospital- and community-acquired UTIs may influence therapeutic recommendations for UTIs based on setting of acquisition.

The prevalence of AMR including hospital and community urinary *E*. *coli* resistance levels is not completely known in Australia. Obtaining this information is important because it not only provides knowledge about the health status of a population, but also contributes to disease management decisions [13]. This study describes the AMR temporal trends and seasonal variation of *E*. *coli* UTI over five years at an Australian tertiary hospital. The study also compares the prevalence of resistance between hospital- and community-acquired *E*. *coli* UTIs.

## Materials and Methods

### Study design and setting

A retrospective cross sectional design was used. The study was conducted with data from ACT Pathology which is based at a tertiary referral hospital, the Canberra Hospital and Health Services. This is Australian Capital Territory’s (ACT) main hospital which provides acute and specialist care services to over 600,000 people in the surrounding region. The 600 bed publicly-funded hospital which includes an emergency department and intensive care unit, offers a comprehensive range of health services such as acute inpatient and day services, outpatient services, women's and children's services and pathology services. Solid organ transplant services are not offered in Canberra.

Human research ethics approval was granted by ACT Health Human Research Ethics Committee’s Low Risk Sub-Committee and Australian Catholic University Human Research Ethics Committee. Consent from patients was not obtained as a waiver of consent was granted by the ethics committees.

### Urine sample and data collection

The microbiology records of inpatients and those attending Canberra Hospital who had urine samples processed at ACT Pathology from January 2009 to December 2013 were retrospectively reviewed. Demographic data and clinical information such as date of birth, gender, admission date, specimen collection date and antimicrobial susceptibility test result were obtained from the microbiology laboratory database and administrative record system.

### Bacterial isolation and identification

Urine samples were analysed and processed based on the microbiology laboratory standards [14]. For this study, a culture with presence of ≥10^7^ colony forming unit (cfu) per litre of urine was considered positive for UTI based on the laboratory recommendations. This 10^7^ cfu/L cut-off is commonly used as it increases the sensitivity of the urine culture test making it a practical threshold [15]. The criterion has also been used by several studies reporting on antimicrobial resistance of urinary *E*. *coli* [1,16,17]. Cultures with three or more bacterial species isolated were considered contaminated and excluded. Only the first positive *E*. *coli* UTI per patient per year was included in the final analysis.

### Definitions

Urine cultures were classified based on the setting of acquisition of infection (hospital-acquired and community-acquired, also known as hospital-onset and community-onset) using criteria from the Centers for Disease Control and Prevention definitions [18]. Positive *E*. *coli* urine cultures obtained within the first 48 hours of admission (including cultures from non-admissions such as outpatient clinics) were defined as community-acquired UTIs. Positive cultures obtained more than 48 hours after admission and within 48 hours of discharge were defined as hospital-acquired UTIs.

### Antimicrobial susceptibility testing

Antimicrobial susceptibility was performed by a disc diffusion method and the automated minimum inhibitory concentration (MIC) method using Vitek2 (Biomerieux Diagnostics). Interpretation was based on Clinical Laboratory Standard Institute (CLSI, formerly NCCLS) criteria [19]. Based on a stepwise laboratory testing protocol used during the study period, all significant *E*. *coli* (>10^7^cfu/L) isolated after overnight incubation on culture had disc susceptibility testing done. The antibiotic discs used for these tests were ampicillin (10μg), amoxycillin-clavulanate (augmentin) (30μg), cephalexin/cefazolin (30 μg), trimethoprim (5μg), nalidixic acid (30 μg), ciprofloxacin (5 μg), nitrofurantoin (300 μg) and gentamicin (10μg). The isolates which were found to be resistant to at least three of the routinely tested antibiotics were then sent for Vitek2 testing to determine the MICs for ceftriaxone, trimethoprim-sulphamethoxazole, meropenem and piperacillin-tazobactam in addition to the routinely tested antibiotics. Direct susceptibility testing method on urine specimens for *E*. *coli* has been validated at ACT Pathology and is comparable to the CLSI recommended methods.

The quality control strains used for disc diffusion tests were *E*. *coli* ATCC 25922, *E*. *faecalis* ATCC 29212 and for Vitek *E*. *coli* ATCC 25922, *P*. *aeruginosa* ATCC 27853, *E*. *faecalis* ATCC 29212, *S*. *aureus* ATCC 29213.

### Extended spectrum beta lactamase (ESBL) confirmation

Detection of ESBL-producing isolates was performed with combination discs of cefotaxime (30μg), cefotaxime/clavulanic acid (30/10μg), ceftazidime (30μg) and ceftazidime/clavulanic acid (30/10μg) whenever required according to CLSI guidelines [15]. Extended spectrum beta lactamase production was inferred when the zone diameter of the disc with clavulanate was ≥5mm larger than the disc without clavulanate for the same antibiotic. *K*. *pneumoniae* ATCC 700603 was used as the quality control strain.

### Statistical analysis

The overall 5-year and yearly resistance rates of *E*. *coli* to the routinely tested first-line antimicrobials on over 4,000 isolates (ampicillin, amoxycillin-clavulanate, cephalexin/cefazolin, ciprofloxacin, gentamicin, nalidixic acid, and trimethoprim) were calculated by dividing the number of urinary *E*. *coli* isolates resistant to each antimicrobial by the number of isolates tested against an individual antimicrobial agent. For the isolates which were sent for further susceptibility testing on Vitek2 against second-line antimicrobials (ceftriaxone, trimethoprim-sulphamethoxazole, meropenem, piperacillin-tazobactam and nitrofurantoin), the denominator used in calculating the resistance rates was the total number of isolates included in the study. This denominator was used based on the assumption that isolates were initially not tested for the Vitek2 antibiotics because they were considered highly unlikely to be resistant to these antibiotics. Hence in order not to overestimate the resistance rates of these isolates the denominator included all isolates tested on both antibiotic discs and Vitek2. The binomial exact 95% confidence intervals (CI) of the resistance percentages were calculated. The 5-year resistance rates were compared for community- and hospital-acquired isolates. The chi-square test was used to check for statistically significant differences in AMR between both groups. Mean differences in age between the two groups were tested using Student’s t-tests. A time series analysis was performed separately for all antimicrobials tested to identify patterns in resistance (trends and seasonal variation) over the five year period. Seasonality is a pattern that shows periodic repetitive fluctuations over time. An autoregressive (AR) model was constructed to assess time-varying resistance patterns (i.e., resistance is non-stationary, or changing, over time) and multiple time series models were fitted to also account for age and sex. The analysis on age and sex followed an ecological study design because these variables were aggregated for each season. The Dickey-Fuller (DF) and the augmented Dickey-Fuller (ADF) tests were used to assess a unit root in the time series data. Both DF and ADF statistics are negative numbers; the more negative, the stronger the rejection of the null hypothesis (that there is unit root at some level of confidence). These unit root tests investigate whether a time series variable (e.g., resistance) is non-stationary using the AR model [20]. Urinary *E*. *coli* isolates for which the antimicrobial showed an intermediate susceptibility category (amoxycillin-clavulanate, trimethoprim, and ciprofloxacin) were excluded from the final analysis. A significance level of *P* < 0.05 was used. Data were analysed using STATA statistical software (version 13, StataCorp).

## Results

A total of 106,512 urine samples from 47,727 patients attending Canberra Hospital from 2009 to 2013 were processed by ACT Pathology. Of these, 14.1% (n = 15,022) had positive cultures with *E*. *coli* being the most common organism isolated in 7670 (51.1%) samples. The distribution of samples by study year is shown in S1 Table.

Of the 7670 *E*. *coli* cultures, most (7103 isolates) could be further classified as community- or hospital-acquired UTI based on available data. The data were then restricted to the first positive *E*. *coli* UTI per patient per year of which there were 5346 positive *E*. *coli* UTIs but only 5333 had susceptibility test results. Hence 5333 *E*. *coli* UTIs belonging to 4732 patients in the 5-year period were included in the final analysis. The majority (84.2%, n = 4492) of UTIs were classified as community-acquired and 15.8% (n = 841) as hospital-acquired. The mean age of all patients was 57.0 years (SD = 27.5) and patients were mostly female (80.2%, n = 3795). There was a significant difference in age between patients with hospital- and community-acquired *E*. *coli* UTI (mean age 67.2 years versus 55.1 years, P<0.001) but no significant differences in gender.

### Antimicrobial resistance

All 5333 isolates had routine susceptibility testing performed against first-line antimicrobials and the overall 5-year and stratified (hospital- and community-acquired) AMR rates are summarised in Table 1. Of the 5333 isolates, 1599 (29.9%) were sent for further antimicrobial susceptibility testing for second-line antimicrobials on Vitek2. The overall 5-year resistance rates to these second-line antimicrobials are reported in Table 2.

**Table 1:** Resistance profile of urinary *E*. *coli* isolates sent for routine susceptibility testing from 2009 to 2013 by setting.

		COMMUNITY			HOSPITAL			TOTAL		
Antibiotic	Year	Number of community isolates tested	R n (%)	95% CI of resistance percentage	Number of hospital isolates tested	R n (%)	95% CI of resistance percentage	Total number of isolates tested*	R n (%)	95% CI of resistance percentage
Ampicillin	2009	835	331 (39.6)	36.3–43.1	143	71 (49.7)	41.2–58.1	978	402 (41.1)	38.0–44.3
	2010	897	358 (39.9)	36.7–43.2	182	70 (38.5)	31.4–45.9	1079	428 (39.7)	36.7–42.7
	2011	1037	443 (42.7)	39.7–45.8	189	91 (48.2)	40.8–55.5	1226	534 (43.6)	40.8–46.4
	2012	939	412 (43.9)	40.7–47.1	173	74 (42.8)	35.3–50.5	1112	486 (43.7)	40.8–46.7
	2013	784	315 (40.2)	36.7–43.7	154	71 (46.1)	38.1–54.3	938	386 (41.2)	38.0–44.4
	Total	4492	1859 (41.4)	39.9–42.8	841	377 (44.8)	41.4–48.3	5333	2236(41.9)	40.6–43.3
AMC	2009	785	24 (3.1)	2.0–4.5	133	6 (4.5)	1.7–9.6	918	30 (3.3)	2.2–4.6
	2010	832	49 (5.9)	4.4–7.7	172	11 (6.4)	3.2–11.2	1004	60 (6.0)	4.6–7.6
	2011	981	61 (6.2)	4.8–7.9	170	17 (10.0)	5.9–15.5	1151	78 (6.8)	5.4–8.4
	2012	895	71 (7.9)	6.2–9.8	161	19 (11.8)	7.3–17.8	1055	89 (8.4)	6.8–10.3
	2013	754	58 (7.7)	5.9–9.8	145	23 (15.9)	10.3–22.8	899	81 (9.0)	7.2–11.1
	Total	4247	263 (6.2)	5.5–6.9	781	76 (9.3)	7.7–12.0	5027	338 (6.7)	6.0–7.5
Cefazolin	2009	821	60 (7.3)	5.6–9.3	129	14 (10.9)	6.1–17.5	950	74 (7.8)	6.2–9.7
	2010	885	96 (10.9)	8.9–13.1	179	24 (13.4)	8.8–19.3	1064	120 (11.3)	9.4–13.3
	2011	1019	103 (10.1)	8.3–12.1	178	30 (16.9)	11.7–23.2	1197	133 (11.1)	9.4–13.0
	2012	917	82 (8.9)	7.2–11.0	168	26 (15.5)	10.4–21.8	1085	108 (10.0)	8.2–11.9
	2013	776	89 (11.5)	9.3–13.9	151	30 (19.9)	13.8–27.1	927	119 (12.8)	10.8–15.2
	Total	4418	430 (9.7)	8.9–10.6	805	124 (15.4)	13.0–18.1	5223	554 (10.6)	9.8–11.5
Trimethoprim	2009	830	153 (18.4)	15.9–21.2	143	28 (19.6)	13.4–27.0	973	181 (18.6)	16.2–21.2
	2010	897	172 (19.2)	16.6–21.9	181	33 (18.2)	12.9–24.6	1078	205 (19.0)	16.7–21.5
	2011	1036	217 (20.9)	18.5–23.6	189	42 (22.2)	16.5–28.8	1225	259 (21.1)	18.9–23.5
	2012	939	200 (21.3)	18.7–24.1	173	40 (23.1)	17.1–30.1	1112	240 (21.6)	19.2–24.1
	2013	784	181 (23.1)	20.2–26.2	154	36 (23.4)	16.9–30.9	938	217 (23.1)	20.5–26.0
	Total	4486	923 (20.6)	19.4–21.8	840	179 (21.3)	18.6–24.2	5326	1102(20.7)	19.6–21.8
Nalidixic acid	2009	826	63 (7.6)	5.9–9.7	143	12 (8.4)	4.4–14.2	969	75 (7.7)	6.1–9.6
	2010	892	73 (8.2)	6.5–10.2	182	12 (6.6)	3.5–11.2	1074	85 (7.9)	6.4–9.7
	2011	1034	109 (10.5)	8.7–12.6	188	22 (11.7)	7.5–17.2	1222	131 (10.7)	9.0–12.6
	2012	755	56 (7.4)	5.7–9.5	140	17 (12.1)	7.2–18.7	895	73 (8.2)	6.4–10.1
	2013	585	33 (5.6)	3.9–7.8	103	11 (10.7)	5.5–18.3	688	44 (6.4)	4.7–8.5
	Total	4092	334 (8.2)	7.3–9.0	756	74 (9.8)	7.8–12.1	4848	408 (8.4)	7.6–9.2
Ciprofloxacin	2009	808	33 (4.1)	2.8–5.7	139	7 (5.0)	2.0–10.1	947	40 (4.2)	3.0–5.7
	2010	701	35 (5.0)	3.5–6.9	150	4 (2.7)	0.7–6.7	851	39 (4.6)	3.3–6.2
	2011	795	52 (6.5)	4.9–8.5	156	10 (6.4)	3.1–11.5	951	62 (6.5)	5.0–8.3
	2012	749	56 (7.5)	5.7–9.6	143	11 (7.7)	3.9–13.3	892	67 (7.5)	5.9–9.4
	2013	631	60 (9.5)	7.3–12.1	135	17 (12.6)	7.5–19.4	766	77 (10.1)	8.0–12.4
	Total	3684	236 (6.4)	5.6–7.2	723	49 (6.8)	5.1–8.9	4407	285 (6.5)	5.8–7.2
Gentamicin	2009	514	17 (3.3)	1.9–5.2	85	5 (5.9)	1.9–13.2	599	22 (3.7)	2.3–5.5
	2010	893	23 (2.6)	1.6–3.8	182	2 (1.1)	0.1–3.9	1075	25 (2.3)	1.5–3.4
	2011	1036	38 (3.7)	2.6–5.0	189	12 (6.4)	3.3–10.8	1225	50 (4.1)	3.0–5.3
	2012	931	40 (4.3)	3.1–5.8	172	12 (7.0)	3.7–11.9	1102	52 (4.7)	3.5–6.1
	2013	783	36 (4.6)	3.2–6.3	154	10 (6.5)	3.2–11.6	937	46 (4.9)	3.6–6.5
	Total	4157	154 (3.7)	3.2–4.3	782	41 (5.2)	3.8–7.0	4938	195 (3.9)	3.4–4.5

*Note that not all 5333 isolates were tested against each antimicrobial. Isolates not tested: AMC = 3; Cephazolin = 110; Trimethoprim = 3; Nalidixic acid = 485; Ciprofloxacin = 893; Gentamicin = 395

Number of isolates with intermediate susceptibility to an antimicrobial: AMC = 303; Trimethoprim = 4; Ciprofloxacin = 33

R = Resistant

n = Number of isolates

AMC = Amoxycillin-clavulanate; TMP-SMX = Trimethoprim-sulphamethoxazole

**Table 2:** Resistance profile of urinary *E*. *coli* isolates sent for further testing on Vitek2 from 2009 to 2013 by setting.

Year	Setting	N	Antibiotic									
			Ceftriaxone		TMP-SMX		MER		PIT		NIT	
			R n (%)	95% CI of resistance percentage	R n (%)	95% CI of resistance percentage	R n (%)	95% CI of resistance percentage	R n (%)	95% CI of resistance percentage	R n (%)	95% CI of resistance percentage
2009	CA	835	12 (1.4)	0.7–2.5	58 (6.9)	5.3–8.9	0 (0.0)	-	2 (0.2)	0.0–0.9	14 (1.7)	0.9–2.8
	HA	143	2 (1.4)	0.2–5.0	12 (8.4)	4.4–14.2	0 (0.0)	-	0 (0.0)	-	4 (2.8)	0.8–7.0
	Total	978	14 (1.4)	0.8–2.4	70 (7.2)	5.6–9.0	0 (0.0)	-	2 (0.2)	0.0–0.7	18 (1.8)	1.1–2.9
2010	CA	897	22 (2.5)	1.5–3.7	76 (8.5)	6.7–10.5	0 (0.0)	-	27 (3.0)	2.0–4.3	15 (1.7)	0.9–2.7
	HA	182	4 (2.2)	0.6–5.5	11 (6.0)	3.1–10.6	0 (0.0)	-	4 (2.2)	0.6–5.5	5 (2.7)	0.9–6.3
	Total	1079	26 (2.4)	1.6–3.5	87 (8.1)	6.5–9.9	0 (0.0)	-	31 (2.9)	2.0–4.1	20 (1.9)	1.1–2.8
2011	CA	1037	46 (4.4)	3.3–5.9	99 (9.5)	7.8–11.5	0 (0.0)	-	19 (1.8)	1.1–2.8	17 (1.6)	1.0–2.6
	HA	189	13 (6.9)	3.7–11.5	30 (15.9)	11.0–21.9	0 (0.0)	-	9 (4.8)	2.2–8.8	3 (1.6)	0.3–4.6
	Total	1226	59 (4.8)	3.7–6.2	129 (10.5)	8.9–12.4	0 (0.0)	-	28 (2.3)	1.5–3.3	20 (1.6)	1.0–2.5
2012	CA	939	43 (4.6)	3.3–6.1	102 (10.9)	8.9–13.0	1 (0.1)	0.0–0.6	33 (3.5)	2.4–4.9	35 (3.7)	2.6–5.1
	HA	173	13 (7.5)	4.1–12.5	22 (12.7)	8.1–18.6	0 (0.0)	-	15 (8.7)	4.9–13.9	5 (2.9)	0.9–6.6
	Total	1112	56 (5.0)	3.8–6.5	124 (11.1)	9.4–13.1	1 (0.1)	0.0–0.5	48 (4.3)	3.2–5.7	40 (3.6)	2.6–4.9
2013	CA	784	45 (5.7)	4.2–7.6	87 (11.1)	9.0–13.5	0 (0.0)	-	30 (3.8)	2.6–5.4	42 (5.4)	3.9–7.2
	HA	154	15 (9.7)	5.6–15.6	25 (16.2)	10.8–23.0	0 (0.0)	-	16 (10.4)	6.1–16.3	4 (2.6)	0.7–6.5
	Total	938	60 (6.4)	4.9–8.2	112 (11.9)	9.9–14.2	0 (0.0)	-	46 (4.9)	3.6–6.5	46 (4.9)	3.6–6.5
Total	CA	4492	168 (3.7)	3.2–4.3	422 (9.4)	8.6–10.3	1 (0.0)	0.0–0.1	111 (2.5)	2.0–3.0	123 (2.7)	2.3–3.3
	HA	841	47 (5.6)	4.1–7.4	100 (11.9)	9.8–14.3	0 (0.0)	-	44 (5.2)	3.8–7.0	21 (2.5)	1.6–3.8
	Total	5333	215 (4.0)	3.5–4.6	522 (9.8)	9.0–10.6	1 (0.0)	0.0–0.1	155 (2.9)	2.5–3.4	144 (2.7)	2.3–3.2

R = Resistant

N = Number of isolates tested

CA = Community isolates; HA = Hospital isolates

TMP-SMX = Trimethoprim-sulphamethoxazole; MER = Meropenem; PIT = Piperacillin-tazobactam; NIT = Nitrofurantoin

**Fig 1:**
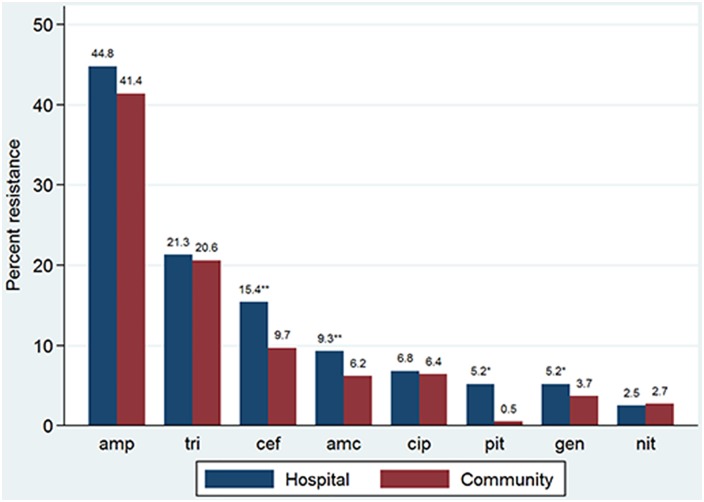
Five-year resistance rates of hospital- and community-acquired *E*. *coli* UTIs by selected antibiotics. amp = ampicillin; tri = trimethoprim; cef = cefazolin; amc = amoxycillin-clavulanate; cip = ciprofloxacin; pit = piperacillin-tazobactam; gen = gentamicin; nit = nitrofurantoin. ** 0.001 < p value < 0.05. ** p < 0.001

The highest overall 5-year resistance rates to urinary *E*. *coli* for both hospital and community isolates combined were seen for ampicillin (41.9%; 95% CI = 40.6–43.3) and trimethoprim (20.7%; 95% CI = 19.6–21.8). The lowest resistance rates were for meropenem (0.0%), nitrofurantoin (2.7%; 95% CI = 2.3–3.2) and piperacillin-tazobactam (2.9%; 95% CI = 2.5–3.4). Resistance to amoxycillin-clavulanate, cephalexin/cefazolin, gentamicin and piperacillin-tazobactam was significantly higher in hospital- compared to community-acquired UTIs (P<0.001, P<0.001, P = 0.043 and P = 0.002, respectively). For ampicillin, trimethoprim, nalidixic acid, ciprofloxacin, ceftriaxone and trimethoprim-sulphamethoxazole, resistance rates were also higher for hospital- compared with community-acquired UTI but this did not reach statistical significance (Fig 1).

**Fig 2:**
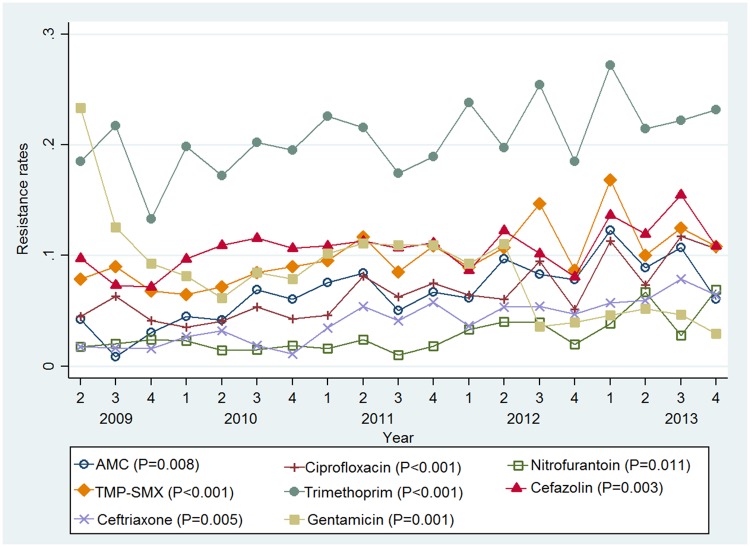
Seasonal antimicrobial resistance rates for *E*. *coli* UTIs. 1 = Summer; 2 = Autumn; 3 = Winter; 4 = Spring. P = significance level for an increasing trend. AMC = Amoxycillin-clavulanate; TMP-SMX = Trimethoprim-sulphamethoxazole

Trend analysis showed a significant increase in resistance to amoxycillin-clavulanate, trimethoprim, ciprofloxacin, nitrofurantoin, trimethoprim-sulphamethoxazole, cefazolin, ceftriaxone and gentamicin over the five year period (Fig 2). There was no significant increase in resistance for ampicillin, nalidixic acid, meropenem and piperacillin-tazobactam. A seasonal pattern was only observed for trimethoprim (ADF statistic = -4.136; P = 0.006) with higher resistance rates for this antimicrobial seen in the summer months. Regression analysis indicated an association between increasing age and resistance to ciprofloxacin (regression coefficient = 0.01; P = 0.004), cefazolin (regression coefficient = 0.004; P = 0.038) and ceftriaxone (regression coefficient = 0.01; P = 0.002).

### ESBL production

Overall 5-year prevalence of ESBL-producing *E*. *coli* isolates was 1.9% (95% CI = 1.5–2.3; n = 100). Extended spectrum beta-lactamase production was low by international standards but was significantly higher in hospital-acquired (3.0%; 95% CI = 1.9–4.4; n = 25) compared with community-acquired UTIs (1.7%; 95% CI = 1.3–2.1; n = 75, P = 0.01). The levels of ESBL-producing *E*. *coli* increased from 0.7% (95% CI = 0.0–3.8) in hospital-acquired UTIs in 2009 to 6.5% (95% CI = 3.2–11.6) in 2013. An increase was also noted for community-acquired UTIs (0.6%; 95% CI = 0.2–1.4 in 2009 to 3.7%; 95% CI = 2.5–5.3 in 2013). The increasing trend in ESBL production over the five years was statistically significant for both hospital (P = 0.035) and community-acquired UTIs (P<0.001).

## Discussion

This study provides information about the AMR pattern of *E*. *coli* UTIs in an Australian tertiary hospital. To our knowledge this is the first Australian study to compare AMR in hospital- and community-acquired *E*. *coli* UTI and assess AMR temporal trends and seasonal variation of *E*. *coli* UTI over time. Our results showed that overall resistance was highest for ampicillin and trimethoprim. We also found significantly higher resistance rates in hospital- compared to community-acquired UTIs for amoxycillin-clavulanate, cephalexin/cefazolin, gentamicin and piperacillin-tazobactam with an increasing resistance trend for eight of the twelve antimicrobials tested which include the four commonly used antimicrobials for first line treatment of UTI in Australia.

In Australia, trimethoprim, cephalexin, amoxycillin-clavulanate or nitrofurantoin are recommended for first line treatment of UTI [21]. The Infectious Diseases Society of America (IDSA) and European Society for Microbiology and Infectious Diseases recommend trimethoprim-sulphamethoxazole as an appropriate treatment choice if local resistance rates do not exceed 20%. The IDSA guidelines also recommend that amoxycillin or ampicillin should not be used alone for empirical treatment because of the relatively poor efficacy and the relatively high prevalence of AMR to these agents worldwide [22]. Given the high levels of resistance to ampicillin and trimethoprim identified in this study, the appropriateness of these antimicrobials in the management of UTI in this patient population should be assessed. The IDSA suggests that beta-lactam agents, including amoxycillin-clavulanate are appropriate choices for therapy when other recommended agents cannot be used [22]. Based on our findings, the majority of UTIs have very low resistance to amoxycillin-clavulanate and nitrofurantoin which are commonly used for UTI treatment in Canberra. Ciprofloxacin, which is recommended in Australia for complicated UTIs, was also found to have a low resistance rate. Through the national pharmaceutical subsidy scheme, the use of quinolones in humans has been restricted in Australia. Quinolone use in food-producing animals is also not permitted. Therefore, fluoroquinolone resistance in the community has been slow to emerge and has remained at low levels in important pathogens such as *E*. *coli* compared to most countries [23]. Our overall resistance rates are also generally lower than reported for other single site studies [9,24], demonstrating that resistance may vary geographically, as shown in a recent meta-analysis [25]. The explanation for the varying resistance rates is not clearly understood but possible reasons have been postulated. A study conducted in the United States demonstrated a geographic gradient in resistance with the highest resistance rates noted in the Pacific region and lowest rates in the South Atlantic region [26]. It was suggested that geographic clustering of resistance phenotypes may have accounted for the geographic differences in resistance. It is therefore possible that the lower rates we found in comparison to those reported for other single site studies may be due to lower levels of bacteria with resistance phenotypes in our locality. Another possible suggestion for geographic variation in resistance is the differences in antimicrobial use [26,27]. Several studies have demonstrated an association between antimicrobial use and resistance [28–30]. Hence it is probable that the lower resistance noted may be as a result of lower antimicrobial use resulting in lower antimicrobial selection pressure. This emphasizes the need for continuous local monitoring of resistance patterns to ensure appropriate treatment for people in the locality.

The sample size of the data was able to detect some significant differences between community- and hospital-acquired UTI resistance rates but for some antimicrobials the differences observed could not be confirmed statistically, possibly due to an insufficient sample size. Overall, we found lower rates of antibiotic resistance for community- compared with hospital-acquired *E*. *coli* UTIs, consistent with other studies [9,31]. The difference in resistance rates is however only small and supports the view that *E*. *coli*, a bacterium carried in the bowel and acquired in the community, is brought into hospital usually by patients themselves rather than being hospital-acquired. This finding may also have been partially dictated by our methodology from using the first positive UTI per person per year. The different resistance rates for hospital- and community-acquired urinary *E*. *coli* isolates seen in this study are comparable with findings reported previously [10,11]. Similar results have been seen in blood culture isolates of *E*. *coli* in Canberra [32]. While the difference in resistance rates was not large and most antimicrobial use occurs in the community, the proportion of patients receiving antimicrobials is much higher in the hospital and hence explains the difference seen [33]. We agree with recommendations that to accurately represent *E*. *coli* resistance rates, antibiograms should be stratified by setting of infection onset [34].

The increasing resistance trend noted in our study for the eight antimicrobials is consistent with previously reported Australian data and published studies from other developed countries [6,7,9,35,36]. The increasing trend may be attributable to antimicrobial overuse or misuse which is a known risk factor for the development of AMR [37]. However, clinical data on hospital antimicrobial use at the study location showed stable rates for most antimicrobials tested (data not shown). We also found seasonal increases in trimethoprim resistance especially in summer months. The literature suggests a possible seasonality with UTI incidence [38,39] but this was not demonstrated in our study. It is possible that seasonality in UTI may lead to seasonal variation in antimicrobial use with subsequent seasonal resistance patterns although to our knowledge, this is yet to be demonstrated in published studies. Evidence currently exists to show higher use of antimicrobials in winter months which is likely related to the increased incidence of respiratory tract infections during that period with consequent increases in resistance during winter [40,41]. Therefore the seasonal trimethoprim resistance is a potentially important finding which should be explored in future studies especially in relation to antimicrobial use. The ecological analysis conducted in this study showed an association between older age and resistance to ciprofloxacin, cefazolin and ceftriaxone consistent with published studies [6,34,42]. The association between increasing age and increased resistance is not surprising given that the physiological changes caused by aging and increased comorbidities predispose to a higher risk of infection leading to more contact with healthcare settings and hence more frequent exposure to antibiotics [42].

It is worth emphasizing that our overall ESBL rate of 1.9% was low compared to most other published studies [31]. Results from the 2009–2011 SMART study in the United States reported an ESBL rate of 6.8% for *E*. *coli* UTI [31]. Although our reported ESBL rate is relatively low, the presence and increasing trend of ESBL-producing *E*. *coli* in both hospital and community-acquired UTIs pose considerable public health concern. This is because this organism renders many of the conventional empirical treatment options for UTI ineffective especially in community-acquired UTI where options for oral antibiotic therapy appear to be limited [43]. For hospital-acquired UTI caused by ESBL-producing *E*. *coli*, carbapenems are considered the treatment of choice [43]. In our study, the lowest resistance rate reported was for meropenem, a carbapenem.

This study has some limitations. As most UTIs are treated empirically, it is possible that samples submitted to the laboratory included patients with recurrent UTIs and asymptomatic bacteriuria thereby overestimating the resistance rates. In addition, inclusion of the first positive *E*. *coli* UTI per person per year may have underestimated the resistance rates reported in our study. Evidence suggests that analysis of antimicrobial resistance data should include each individual positive isolate in order to ensure sensitivity, but this positive isolate should only be included once to guarantee specificity [44]. This approach of using only the first positive isolate per patient per year is also consistent with published studies on resistance in UTI pathogens including *E*. *coli* [17,34]. It is unlikely that repeated isolates are correlated but there is a small possibility that this could occur although it was not accounted for in the analysis. The 5-year period prevalence study could therefore have overestimated the resistance. The use of routinely collected microbiology data also posed some limitations as clinical information on patients including comorbidities and presence of indwelling urethral catheters was often missing. The incompleteness of this information prevented its inclusion in the analysis. This study was based on retrospective antimicrobial susceptibility data from a National Association of Testing Authorities, Australia (NATA) accredited clinical microbiology laboratory. The stepwise laboratory testing protocol involved routine first-line antibiotic sensitivity testing followed by more extensive testing with second-line antibiotics only for isolates resistant to at least three of the routine antibiotics. Although this laboratory approach is widely used [44] there is the potential for testing bias and/or selection bias with consequent overestimation of resistance rates. Given the lack of consensus on an appropriate denominator using this testing approach and to prevent possible overestimation of the resistance rates against second-line antibiotics, the denominator therefore included all isolates tested, which, in turn, may have under-estimated resistance rates of broad spectrum antimicrobials. Determining the resistance rate can be influenced by the extent of laboratory testing which in turn influences the selection of the denominator. Using the total number of isolates tested or the number of isolates tested against second-line antibiotics alone as the denominator will either underestimate or overestimate the resistance rates respectively. Although using all isolates for calculating resistance rates for second-line antibiotics has its limitations, this was an appropriate denominator choice to make the findings relevant for use in the clinical setting. For ideal comparison of susceptibility patterns, all isolates would need to be tested against the extended panel of antibiotics in a properly designed prospective study. Regardless of these limitations, our reported resistance rates are low compared to other studies. The use of ecological data to account for the effects of age and sex on resistance also poses limitations to interpretation of these results at the individual patient level. Although our data are from a single tertiary hospital and may not be generalisable to other populations, the data were reported by a NATA accredited laboratory and are therefore satisfactory to provide recommendations to guide local empirical therapy.

## Conclusions

Antimicrobial resistance poses grave concerns for antimicrobial effectiveness in treating infections such as UTI. This study demonstrates the increasing resistance of urinary *E*. *coli* to commonly prescribed antimicrobials. Amoxycillin-clavulanate and nitrofurantoin are still effective for empirical treatment of UTI in this population. Overuse of ampicillin and trimethoprim should be avoided given the high resistance rates reported. In developing local antimicrobial prescribing guidelines, the choice of antimicrobial in the treatment of UTI should be based on setting (community or hospital) of acquisition.

## Supporting Information

S1 Table Distribution of all Canberra Hospital urine samples from 2009 to 2013. (DOC)

## References

[1] Laupland K, Ross T, Pitout J, Church D, Gregson D. Community-onset urinary tract infections: a population-based assessment. Infection 2007;35:150–3. 10.1007/s15010-007-6180-2 17565455

[2] Nicolle LE. Uncomplicated urinary tract infection in adults including uncomplicated pyelonephritis. Urologic Clinics of North America 2008;35:1–12. 10.1016/j.ucl.2007.09.004 18061019

[3] Centers for Disease Control and Prevention, National Center for Health Statistics. 2009–2010 Combined Year Tables-Combined NAMCS and NHAMCS. USA: Centers for Disease Control and Prevention 2015 Available: http://www.cdc.gov/nchs/data/ahcd/combined_tables/2009-2010_combined_web_table01.pdf

[4] Mitchell BG, Fasugba O, Beckingham W, Bennett N, Gardner A. A point prevalence study of healthcare associated urinary tract infections in Australian acute and aged care facilities. Infection, Disease & Health 2016;21:26–31. 10.1016/j.idh.2016.03.001

[5] Howard DH, Scott RD, Packard R, Jones D. The global impact of drug resistance. Clinical Infectious Diseases 2003;36:S4–10. 10.1086/344656 12516025

[6] Blaettler L, Mertz D, Frei R, Elzi L, Widmer A, Battegay M, et al Secular trend and risk factors for antimicrobial resistance in *Escherichia coli* isolates in Switzerland 1997–2007. Infection 2009;37:534–9. 10.1007/s15010-009-8457-0 20013094

[7] Australian Group for Antimicrobial Resistance. 2011 Antimicrobial susceptibility report: Gram-negative Survey. 2012. Australian Group for Antimicrobial resistance, Australia.

[8] Karlowsky JA, Kelly LJ, Thornsberry C, Jones ME, Sahm DF. Trends in antimicrobial resistance among urinary tract infection isolates of *Escherichia coli* from female outpatients in the United States. Antimicrobial Agents and Chemotherapy 2002;46:2540–5. 10.1128/AAC.46.8.2540-2545.2002 12121930 PMC127340

[9] Cullen IM, Manecksha RP, McCullagh E, Ahmad S, O'Kelly F, Flynn RJ, et al The changing pattern of antimicrobial resistance within 42,033 *Escherichia coli* isolates from nosocomial, community and urology patient-specific urinary tract infections, Dublin, 1999–2009. BJU International 2012;109:1198–206. 10.1111/j.1464-410X.2011.10528.x 21883861

[10] Ma KL, Wang CX. Analysis of the spectrum and antibiotic resistance of uropathogens in vitro: Results based on a retrospective study from a tertiary hospital. American Journal of Infection Control 2013;41:601–6. 10.1016/j.ajic.2012.09.015 23352074

[11] Perrin M, Donnio P, Heurtin-Lecorre C, Travert M, Avril J-L. Comparative antimicrobial resistance and genomic diversity of *Escherichia coli* isolated from urinary tract infections in the community and in hospitals. Journal of Hospital Infection 1999;41:273–9. 10.1053/jhin.1998.0521 10392333

[12] Coxeter P, Looke D, Hoffmann T, Lowe J, Del Mar C. The antibiotic crisis: charting Australia's path towards least resistance. Australian and New Zealand Journal of Public Health 2013;37:403–4. 10.1111/1753-6405.12137 24090320

[13] Buttner P, Muller R. Epidemiology. Victoria, Australia: Oxford University Press; 2011.

[14] Australian Capital Territory Pathology. Microbiology Department Urine Manual. Canberra, Australia; 2013.

[15] Wilson ML, Gaido L. Laboratory Diagnosis of Urinary Tract Infections in Adult Patients. Clinical infectious diseases 2004;38:1150–58. 10.1086/383029 15095222

[16] Linhares I, Raposo T, Rodrigues A, Almeida A. Frequency and antimicrobial resistance patterns of bacteria implicated in community urinary tract infections: a ten-year surveillance study (2000–2009). BMC Infectious Diseases 2013;13 10.1186/1471-2334-13-19 23327474 PMC3556060

[17] McGregor JC, Elman MR, Bearden DT, Smith DH. Sex-and age-specific trends in antibiotic resistance patterns of *Escherichia coli* urinary isolates from outpatients. BMC family practice 2013;14 10.1186/1471-2296-14-25 23433241 PMC3610120

[18] Horan TC, Andrus M, Dudeck MA. CDC/NHSN surveillance definition of health care—associated infection and criteria for specific types of infections in the acute care setting. American Journal of Infection Control 2008;36:309–32. 10.1016/j.ajic.2008.03.002 18538699

[19] Clinical and Laboratory Standards Institute. Performance Standards for Antimicrobial Susceptibility Testing; Twenty-Fourth Informational Supplement. CLSI document M100-S24.Pennsylvania, USA; 2014.

[20] Dickey DA, Fuller WA. Distribution of the estimators for autoregressive time series with a unit root. Journal of the American Statistical Association 1979;74:427–31. 10.1080/01621459.1979.10482531

[21] Antibiotic Expert Groups. Therapeutic guidelines: antibiotic. Version 15 Melbourne: Therapeutic Guidelines Limited; 2014.

[22] Gupta K, Hooton TM, Naber KG, Wullt B, Colgan R, Miller LG, et al International clinical practice guidelines for the treatment of acute uncomplicated cystitis and pyelonephritis in women: a 2010 update by the Infectious Diseases Society of America and the European Society for Microbiology and Infectious Diseases. Clinical Infectious Diseases 2011;52:e103–e20. 10.1093/cid/ciq257 21292654

[23] Cheng AC, Turnidge J, Collignon P, Looke D, Barton M, Gottlieb T. Control of fluoroquinolone resistance through successful regulation, Australia. Emerging Infectious Diseases 2012;18:1453–60. 10.3201/eid1809.111515 22932272 PMC3437704

[24] Wang Y, Zhao S, Han L, Guo X, Chen M, Ni Y, et al Drug resistance and virulence of uropathogenic *Escherichia coli* from Shanghai, China. The Journal of Antibiotics 2014;67:799–805. 10.1038/ja.2014.72 24984795

[25] Fasugba O, Gardner A, Mitchell BG, Mnatzaganian G. Ciprofloxacin resistance in community-and hospital-acquired *Escherichia coli* urinary tract infections: a systematic review and meta-analysis of observational studies. BMC Infectious Diseases 2015;15:1 10.1186/s12879-015-1282-4 26607324 PMC4660780

[26] Sannes MR, Kuskowski MA, Johnson JR. Geographical distribution of antimicrobial resistance among *Escherichia coli* causing acute uncomplicated pyelonephritis in the United States. FEMS Immunology & Medical Microbiology 2004;42:213–18. 10.1016/j.femsim.2004.05.004 15364106

[27] Gupta K, Sahm DF, Mayfield D, Stamm WE. Antimicrobial resistance among uropathogens that cause community-acquired urinary tract infections in women: a nationwide analysis. Clinical infectious diseases 2001;33:89–94. 10.1086/320880 11389500

[28] Bergman M, Nyberg ST, Huovinen P, Paakkari P, Hakanen AJ, et al Association between Antimicrobial Consumption and Resistance in *Escherichia coli*. Antimicrobial Agents and Chemotherapy 2009;53:912–917. 10.1128/AAC.00856-08 19104012 PMC2650536

[29] Goossens H, Ferech M, Vander Stichele R, Elseviers M. Outpatient antibiotic use in Europe and association with resistance: a cross-national database study. The Lancet 2005;365:579–87. 10.1016/S0140-6736(05)17907-0 15708101

[30] Vellinga A, Murphy AW, Hanahoe B, Bennett K, Cormican M. A multilevel analysis of trimethoprim and ciprofloxacin prescribing and resistance of uropathogenic *Escherichia coli* in general practice. Journal of Antimicrobial Chemotherapy 2010;65:1514–20. 10.1093/jac/dkq149 20457673

[31] Bouchillon SK, Badal RE, Hoban DJ, Hawser SP. Antimicrobial susceptibility of inpatient urinary tract isolates of Gram-negative bacilli in the United States: Results from the Study for Monitoring Antimicrobial Resistance Trends (SMART) Program: 2009− 2011. Clinical Therapeutics 2013;35:872–7. 10.1016/j.clinthera.2013.03.022 23623624

[32] Kennedy KJ, Roberts JL, Collignon PJ. *Escherichia coli* bacteraemia in Canberra: incidence and clinical features. Medical Journal of Australia 2008;188:209–13. 18279126 10.5694/j.1326-5377.2008.tb01586.x

[33] Zarb P, Coignard B, Griskeviciene J, Muller A, Vankerckhoven V, Weist K, et al The European Centre for Disease Prevention and Control (ECDC) pilot point prevalence survey of healthcare-associated infections and antimicrobial use. Eurosurveillance 2012;17: pii = 20316. http://www.eurosurveillance.org/ViewArticle.aspx?ArticleId=20316 23171822 10.2807/ese.17.46.20316-en

[34] Swami SK, Liesinger JT, Shah N, Baddour LM, Banerjee R. Incidence of antibiotic-resistant *Escherichia coli* bacteriuria according to age and location of onset: a population-based study from Olmsted County, Minnesota. Mayo Clinic Proceedings 2012;87:753–9. 10.1016/j.mayocp.2012.02.025 22795635 PMC3538489

[35] Hsu L-Y, Tan T-Y, Tam VH, Kwa A, Fisher DA, et al Surveillance and correlation of antibiotic prescription and resistance of Gram-negative bacteria in Singaporean hospitals. Antimicrobial Agents and Chemotherapy 2010;54:1173–78. 10.1128/AAC.01076-09 20065055 PMC2826017

[36] Wong PH, von Krosigk M, Roscoe DL, Lau TT, Yousefi M, et al Antimicrobial co-resistance patterns of gram-negative bacilli isolated from bloodstream infections: a longitudinal epidemiological study from 2002–2011. BMC infectious diseases 2014;14 10.1186/1471-2334-14-393 25308184 PMC4287581

[37] Costelloe C, Metcalfe C, Lovering A, Mant D, Hay AD. Effect of antibiotic prescribing in primary care on antimicrobial resistance in individual patients: systematic review and meta-analysis. BMJ 2010;340:c2096 10.1136/bmj.c2096 20483949

[38] Freeman J, Anderson D, Sexton D. Seasonal peaks in *Escherichia coli* infections: possible explanations and implications. Clinical Microbiology and Infection 2009;15:951–3. 10.1111/j.1469-0691.2009.02866.x 19845705

[39] Perencevich EN, McGregor JC, Shardell M, Furuno JP, Harris AD, et al Summer peaks in the incidences of gram-negative bacterial infection among hospitalized patients. Infection Control & Hospital Epidemiology 2008;29:1124–31. 10.1086/592698 19031546

[40] Dagan R, Barkai G, Givon-Lavi N, Sharf AZ, Vardy D, et al Seasonality of Antibiotic-Resistant Streptococcus pneumoniae That Causes Acute Otitis Media: A Clue for an Antibiotic-Restriction Policy? The Journal of infectious diseases 2008;197:1094–1102. 10.1086/528995 18419528 PMC2652754

[41] Sun L, Klein EY, Laxminarayan R. Seasonality and Temporal Correlation between Community Antibiotic Use and Resistance in the United States. Clinical infectious diseases 2012;55:687–94. 10.1093/cid/cis509 22752512

[42] Adam HJ, Baxter MR, Davidson RJ, Rubinstein E, Fanella S, Karlowsky JA, et al Comparison of pathogens and their antimicrobial resistance patterns in paediatric, adult and elderly patients in Canadian hospitals. Journal of Antimicrobial Chemotherapy 2013;68:i31–i7. 10.1093/jac/dkt024 23587776

[43] Falagas M, Karageorgopoulos D. Extended-spectrum β-lactamase-producing organisms. Journal of Hospital infection 2009;73:345–54. 10.1016/j.jhin.2009.02.021 19596491

[44] Cornaglia G, Hryniewicz W, Jarlier V, Kahlmeter G, Mittermayer H, Stratchounski L, et al European recommendations for antimicrobial resistance surveillance. Clinical Microbiology and Infection 2004;10:349–83. 10.1111/j.1198-743X.2004.00887.x 15059129

