# Supplementary material for: Five-Year Antimicrobial Resistance Patterns of Urinary Escherichia coli at an Australian Tertiary Hospital: Time Series Analyses of Prevalence Data
Source: PLoS One. 2016 Oct 6;11(10):e0164306. doi: 10.1371/journal.pone.0164306 (PMC5053592; doi:10.1371/journal.pone.0164306)
Supplement: S1 Table — (DOC) [file pone.0164306.s001.doc]

**S1 Table**. Distribution of all Canberra Hospital urine samples from 2009 to 2013

|  | 2009 | 2010 | 2011 | 2012 | 2013 | Overall five year period |
| --- | --- | --- | --- | --- | --- | --- |
| Total number of urine samples | 19,455 | 20,005 | 22,070 | 22,496 | 22,486 | 106,512 |
| Proportion of positive urine samples | 14.2%  (n=2,767) | 14.8%  (n=2,951) | 14.3%  (n=3,167) | 13.7%  (n=3,075) | 13.6%  (n=3,062) | 14.1%  (n=15,022) |
| Proportion of positive samples where *E. coli* was isolated | 49.5% (n=1,370) | 50.6% (n=1,494) | 53.6% (n=1,699) | 49.6% (n=1,526) | 51.6% (n=1,581) | 51.1% (n=7,670) |
